# Tolerance of Gambian Plasmodium falciparum to Dihydroartemisinin and Lumefantrine Detected by *Ex Vivo* Parasite Survival Rate Assay

**DOI:** 10.1128/AAC.00720-20

**Published:** 2020-12-16

**Authors:** Haddijatou Mbye, Fatoumata Bojang, Aminata Seedy Jawara, Bekai Njie, Nuredin Ibrahim Mohammed, Joseph Okebe, Umberto D’Alessandro, Alfred Amambua-Ngwa

**Affiliations:** a West African Centre for Cell Biology of Infectious Pathogens, Department of Biochemistry, Cell and Molecular Biology, University of Ghana, Legon, Ghana; b MRC Unit The Gambia at London School of Hygiene and Tropical Medicine, Fajara, The Gambia; c Department of International Public Health, Liverpool School of Tropical Medicine, United Kingdom; d London School of Hygiene and Tropical Medicine, London, United Kingdom

**Keywords:** *Plasmodium falciparum*, drug tolerance, flow cytometry, *ex vivo*, antimalarial agents, artemisinin combination therapies, drug resistance, *ex vivo* survival rates, genotypes

## Abstract

Monitoring of Plasmodium falciparum sensitivity to antimalarial drugs in Africa is vital for malaria elimination. However, the commonly used *ex vivo*/*in vitro* 50% inhibitory concentration (IC_50_) test gives inconsistent results for several antimalarials, while the alternative ring-stage survival assay (RSA) for artemisinin derivatives has not been widely adopted. Here, we applied an alternative two-color flow cytometry-based parasite survival rate assay (PSRA) to detect *ex vivo* antimalarial tolerance in P. falciparum isolates from The Gambia. The PSRA infers parasite viability by quantifying reinvasion of uninfected cells following 3 consecutive days of drug exposure (10-fold the IC_50_ of drug for field isolates). The drug survival rate is obtained for each isolate from the slope of the growth/death curve. We obtained parasite survival rates of 41 isolates for dihydroartemisinin (DHA) and lumefantrine (LUM) out of 51 infections tested by ring-stage survival assay (RSA) against DHA. We also determined the genotypes for known drug resistance genetic loci in the P. falciparum genes *Pfdhfr*, *Pfdhps*, *Pfmdr*, *Pfcrt*, and *Pfk13*. The PSRA results determined for 41 Gambian isolates showed faster killing and lower variance after treatment with DHA than after treatment with LUM, despite a strong correlation between the two drugs. Four and three isolates were tolerant to DHA and LUM, respectively, with continuous growth during drug exposure. Isolates with the PfMDR1-Y184F mutant variant showed increased LUM survival, though the results were not statistically significant. Sulfadoxine/pyrimethamine (SP) resistance markers were fixed, while all other antimalarial variants were prevalent in more than 50% of the population. The PSRA detected *ex vivo* antimalarial tolerance in Gambian P. falciparum. This calls for its wider application and for increased vigilance against resistance to artemisinin combination therapies (ACTs) in this population.

## Abstract (precis)

Monitoring of Plasmodium falciparum sensitivity to antimalarial drugs in Africa is vital for malaria elimination. However, the commonly used *ex vivo*/*in vitro* 50% inhibitory concentration (IC_50_) test gives inconsistent results for several antimalarials, while the alternative ring-stage survival assay (RSA) for artemisinin derivatives has not been widely adopted. Here, we applied an alternative two-color flow cytometry-based parasite survival rate assay (PSRA) to detect *ex vivo* antimalarial tolerance in P. falciparum isolates from The Gambia.

## INTRODUCTION

There has been a substantial decline in malaria morbidity and mortality in sub-Saharan Africa (sSA) over the past decade (1). This decline was mainly driven by the scaling up of control interventions such as long-lasting insecticidal nets and clinical case management with artemisinin combination therapy (ACT) (1). Currently, ACTs are used in countries in regions of endemicity for the treatment of clinical malaria, specifically, for individual chemoprevention in mass treatment campaigns (2). These interventions increase pressure on the parasites, which could result in the emergence of resistance to both partner drugs and to artemisinin derivatives as confirmed previously in Southeast Asia (3, 4) and in reports of delayed parasite clearance in Africa (5).

Currently, the WHO recommends regular efficacy testing of the locally used antimalarials in humans, complemented by *in vitro* (laboratory-based) assessment of parasite growth in response to drug exposure (6). Comparing the *in vitro* efficacies of ACTs is complex as the components have different mechanisms of action (5). Moreover, most of the existing drug susceptibility assays were developed when treatment was based on monotherapies (7, 8). The most common assays are based on the 50% inhibitory concentrations (IC_50_s) required to inhibit parasite growth by half, under a set of experimental conditions (9, 10). This approach is sensitive to variations in the drug concentrations used with inconsistency in data analysis (11). IC_50_ assays also do not assess the temporal course of parasite viability following exposure and are not suited to use with artemisinin derivatives with characteristically shorter half-lives (9).

New *in vitro* methods assessing the efficacy of fast-acting drugs such as the ring-stage survival assay (RSA) (12), the piperaquine survival assay (PSA) (13), and the parasite viability fast assay (PVFA) (14) are now available. RSA and PSA determine parasite survival following drug exposure and withdrawal, while the PVFA aims at discriminating fast-acting antimalarial drugs by assessing parasite killing kinetics over time. There are still critical gaps in these assays. The RSA was designed solely for analysis of fast-acting drugs and therefore cannot be used for analysis of slow-acting antimalarials with longer half-lives (15). The PVFA has been used only in antimalarial development for screening candidate drugs.

Besides *in vitro* assessment of drugs, molecular surveillance is recommended to monitor the emergence and spread of resistance by determining the proportion of isolates in a given population with resistance-associated alleles (16, 17). While the kelch 13 molecular markers of artemisinin resistance have not been identified in sSA (18), alleles in PfCRT and PfMDR1 showing resistance to both currently used and previously used drugs, including partners in ACTs, are in circulation (19). For instance, the use of lumefantrine (LUM) in the ACT artemether-lumefantrine (AL) has been associated with an increase in copy numbers, the frequency of N86 allele, and the N86/184F/D1246 haplotype of PfMDR1 (20–25). Additionally, sulfadoxine/pyrimethamine (SP) is used in seasonal malaria chemoprevention (SMC) and as an intermittent preventive treatment in pregnancy (IPTp), selecting for mutant PfDHFR and PfDHPS alleles (26). Combining molecular surveillance with *in vitro* surveillance can therefore provide an early warning signal indicating the emergence of drug-tolerant parasites. This is critical for a parasite population that is exposed to substantial pressure represented by drug and vector control interventions such as in The Gambia, where malaria transmission and prevalence are low to very low. The Gambia together with neighboring Senegal, is driving for malaria elimination by deploying SMC, while mass drug treatments with ACTs are being contemplated.

Therefore, the goal of this study was to evaluate a parasite survival rate assay (PSRA) to estimate the *ex vivo* drug sensitivity of P. falciparum from The Gambia to the currently used ACT (AL). The PSRA mimics 3 days of exposure to an ACT and determines parasite survival rates over this period. The assay assesses the survival and reinvasion potential of parasites following exposure to LUM and dihydroartemisinin (DHA), which are prototypes of the slow- and fast-acting components of ACTs used in most countries where malaria is endemic. The approach offers significant advantages over the standard IC_50_ determination assay due to its higher sensitivity in measuring parasite viability based on the production of invasive merozoites after drug exposure, representing an index of drug susceptibility or tolerability.

## RESULTS

Plasmodium falciparum isolates collected from patients with uncomplicated malaria cases recruited across the malaria transmission season in 2017 from Western Gambia were analyzed. A total of 79 of 170 (46.5%) isolates had a parasitemia level of ≥0.5%, and these were set up for both PSRA and RSA. Analysis data were obtained for 41 (52%) and 51 (64.6%) samples, which had a drug-free *ex vivo* growth rate of ≥1% for PSRA and RSA, respectively. Apart from the field isolates used in this study, the PSRA was tested against a panel of previously characterized isolates, including an artemisinin-resistant parasite line: MR4-1241 with the K13 I543T mutation.

### Ring stage survival rates of field isolates by microscopy and flow cytometry.

**FIG 1:**
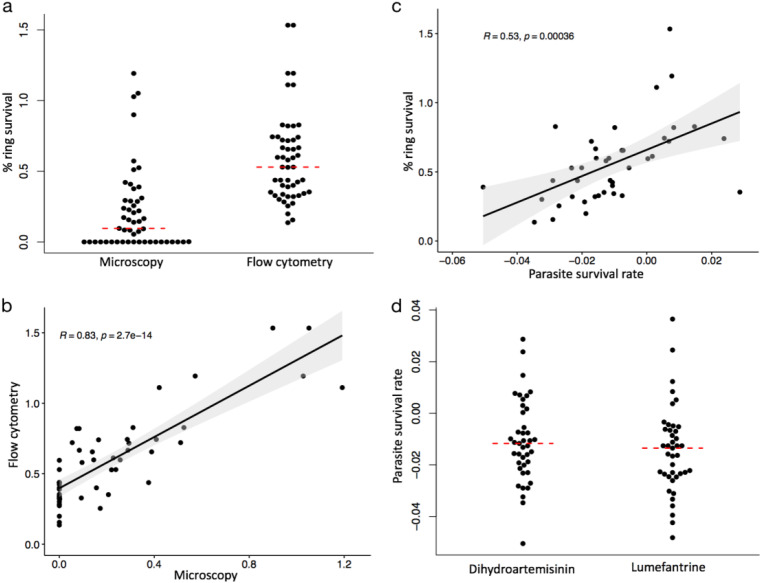
(a) Percent ring survival of 51 isolates determined using conventional microscopy to assess viable parasites and flow cytometry to assess the number of reinvaded parasites following pulse exposure and withdrawal of DHA with RSA. Each point on the plot represents an isolate. The median survival rates of the isolates for each method are shown as the red broken lines. *t* test statistics gave a *P* value of <0.0001 using the Wilcoxon rank sum test. (a and b) Correlation analysis of percent ring survival determined using flow cytometry and microscopy with a Pearson correlation coefficient of *R = *0.83 and a *P* value of <0.0001 (b) and correlation analysis of percent ring survival using RSA and parasite survival rates using PSRA (c). The Pearson correlation coefficient gave an *R* value of 0.53 and a *P* value of 0.00036. (d) Distribution of the parasite survival rates of 41 isolates treated with DHA and LUM at 3 time points over 72 h with PSRA. Each point shows the rate at which each isolate survived following drug exposure with reference to a DMSO-treated control. The red dotted lines represent the median survival rates for both drug treatments with *P = *0.35. A *P* value of <0.05 represents statistical significance. All *ex vivo* assays were performed in triplicate.

Ring-stage survival rates of 51 isolates were determined using conventional microscopy per the initial RSA protocol (12), which was modified here by the use of uninfected red blood cells (uRBC) prelabeled with 7-hydroxy-9H-(1,3-dichloro-9, 9-dimethylacridin-2-one) succinimidyl ester (DDAO-SE) (uRBC^DDAO-SE^) and SYBR green I for flow cytometric analysis. Following pulse exposure to DHA, 31 isolates (61%) had surviving parasites observed by microscopy at levels ranging from 0.05% to 1.2% (Fig. 1a). Flow cytometric counting of reinvasion in prelabeled uRBCs was more sensitive, showing all isolates to have post-drug exposure survival rates that ranged from 0.14% to 1.53%. The mean survival rates determined by flow cytometric analysis were significantly higher than those determined by microscopy (*P* < 0.0001). Despite this, there was a strong positive correlation between the two analysis methods (*R *= 0.83, *P = *2.7 × 10^−14^) (Fig. 1b). However, the isolates with the highest ring survival rates as determined by flow cytometry were not the same as those observed by microscopy. Based on flow cytometry only, percent ring survival after 6 h of exposure to 700 nM DHA significantly correlated with parasite survival rates following PSRA (*R *= 0.53, *P = *0.00038) (Fig. 1d). Overall, the median cumulative rates of survival over the 72 h of exposure were not significantly different between DHA (−0.051% to 0.029%) and LUM (−0.048% to 0.037%) (*P = *0.35), though the responses to LUM had a wider distribution (Fig. 1d).

### P. falciparum *ex vivo* survival decreases with longer drug exposure.

**FIG 2:**
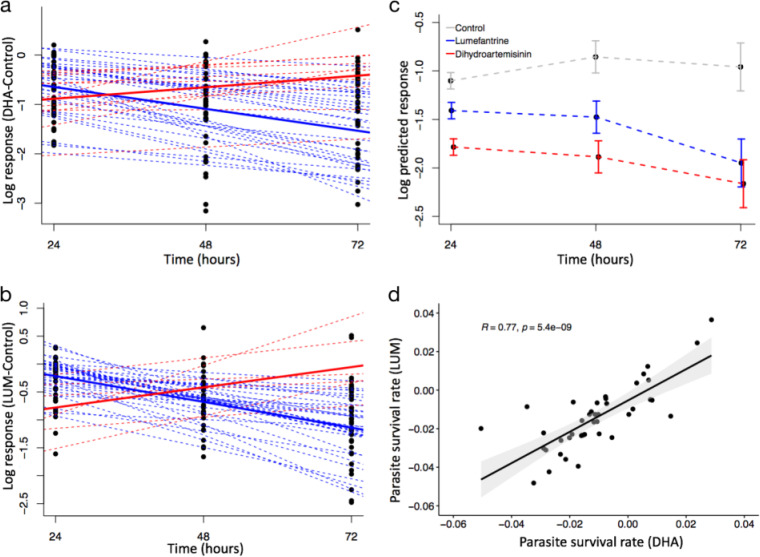
(a and b) Individual trajectories of 41 isolates following exposure to (a) DHA and (b) LUM relative to DMSO-treated controls at the 24-, 48-, and 72-h time points. A linear mixed-effect model was used, and a linear trend was fitted for each isolate across time points. The dotted blue and red lines show the isolates with decreasing and increasing responses over time, respectively. The thick blue and red lines represent the mean log responses of isolates with decreasing and increasing responses, respectively. (c) Mean predicted parasite responses of all isolates following exposure to DHA (red broken line), LUM (blue broken line), and DMSO control (gray broken line), with the standard errors of the means (SEM) shown as bars. (d) Correlation between parasite survival rates of isolates treated with DHA and LUM with *R* = 0.77 and a *P* value of <0.001. All *ex vivo* assays were performed in triplicate.

Comparing log values of survival rates between isolates with different durations of drug exposure, the overall survival rate declined with increased exposure time for both drugs, whereas there was an increasing growth trend in the drug-free group over time (Fig. 2). The mean differences between the treatment and control groups were always statistically significant and increased with time, as the treatment groups appeared to show a marked decline in predicted survival, particularly after 72 h (see Fig. S1 in the supplemental material). Pairwise comparison between the drug-treated groups and the drug-free group showed significant differences at all three time points (Table 1). Using 24 h as the reference, differences in predicted responses were seen for both DHA and LUM at 72 h after drug exposure. The predicted responses seen at 48 h were not statistically significantly different from those seen at 24 h. This could have been due to the exponential increase in the levels of merozoite-infected RBCs seen following a complete P. falciparum growth cycle (27), potentially resulting in the high response levels seen at 48 h in the control group (Fig. 2c).

**TABLE 1:** Effect of drug exposure on predicted responses of the treatment groups (DHA, LUM, and DMSO control) and exposure times (24, 48, and 72 h) for P. falciparum isolates analyzed by PSRA^*a*^

Treatment group	Difference (95% CI)	*P* value
DHA vs control		
24 h	−0.68 (−0.90, −0.47)	**<0.0001**
48 h	−1.03(−1.25, −0.81)	**<0.0001**
72 h	−1.20 (−1.42, −0.99)	**<0.0001**
24 h	−0.31 (−0.52, −0.09)	**0.005**

LUM vs control		
48 h	−0.62 (−0.84, −0.41)	**<0.0001**
72 h	−0.99 (−1.21, −0.77)	**<0.0001**

48 h vs 24 h		
Control	0.25 (0.01, 0.48)	**0.04**
DHA	−0.10 (−0.33, 0.13)	0.39
LUM	0.07 (−0.30, 0.16)	0.57
72 h vs 24 h		
Control	0.14 (−0.13, 0.42)	0.31
DHA	−0.38 (−0.65, −0.10)	**0.007**
LUM	−0.54 (−0.81, −0.27)	**0.0001**

a CI, confidence interval; DHA, dihydroartemisinin treatment; LUM, lumefantrine treatment; control, DMSO treatment. Bold values indicate *P* values representing statistically significant results determined by pairwise comparisons.

### Distribution of PSRA sensitivities to AL.

We derived individual responses to each drug by fitting a linear model to the differences in predicted responses between the drug-treated group and the control group with time. These *ex vivo* parasite survival rates ranged from −0.051 to 0.029 for DHA and from −0.048 to 0.037 for LUM. The majority of isolates had a negative slope with consistently reducing survival with time (Fig. 2a and b). This was seen for 30 isolates for DHA and 35 for LUM, representing 73% and 85% of treated isolates, respectively. In contrast, 27% (11/41) and 15% (6/41) had a net increase in growth despite 72 h of exposure to DHA and LUM, with predicted responses under drug conditions similar to or higher than those determined for the controls treated with dimethyl sulfoxide (DMSO) (Fig. 2a and b; see also Fig. S2a and b). The overall responses and rates of growth decline were higher for DHA than for LUM (Fig. 2c). However, the survival rates seen with DHA and LUM treatment showed a strong positive correlation (*R *= 0.77, *P = *5.4e−09), (Fig. 2d).

### Consistent clusters of survival rate patterns corresponding to both DHA and LUM.

**FIG 3:**
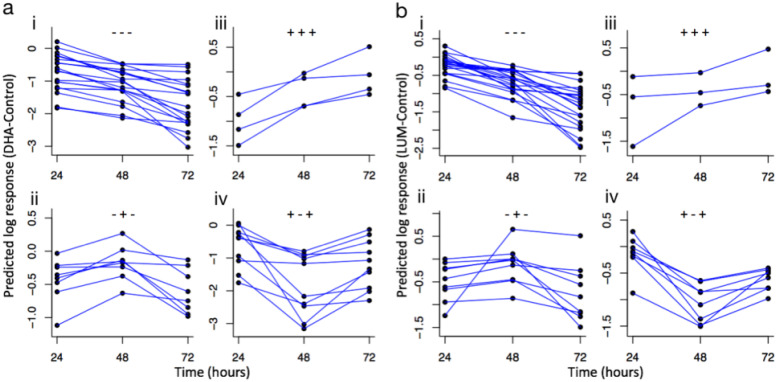
Grouped profiles of 41 isolates following exposure to DHA and LUM at 24, 48, and 72 h with PSRA. Each point in the individual plots represent the difference between the predicted response of the (a) DHA-treated and control isolates and (b) LUM-treated and control isolates. The connecting lines give an indication of the response pattern of each isolate. The isolates are grouped based on their response profiles. (i) Linear decrease (−−−). (ii) Nonlinear decrease/increase (−+−). (iii) Linear increase (+++). (iv) Nonlinear increase/decrease (+−+).

We identified four patterns of responses based on the growth-versus-time curve for both drugs (Fig. 3). The most common pattern was a continuous decline in survival with an increase in time of exposure. This first group of isolates, defined as representing linear decrease (“−−−” in Fig. 3), represented 46% (19/41) and 51% (21/41) of isolates tested against DHA and LUM, respectively. The second group of isolates had a peak in growth at 48 h of drug exposure (“−+−“), and these represented 19.5% (8/41) and 22% (9/41) of isolates tested, respectively. The third group consisted of isolates with consistently linear increases (“+++”) despite drug exposure, with 9.75% (4/41) and 7.3% (3/41) identified for DHA and LUM, respectively, and the fourth pattern consisted of isolates with the lowest survival time point at 48 h (“+−+”), representing 24% (10/41) and 20% (8/41) of isolates, respectively. These patterns did not correlate with initial parasitemia (Fig. S4) or with other patient demographic information.

### Frequencies of drug resistance alleles in western Gambia.

**FIG 4:**
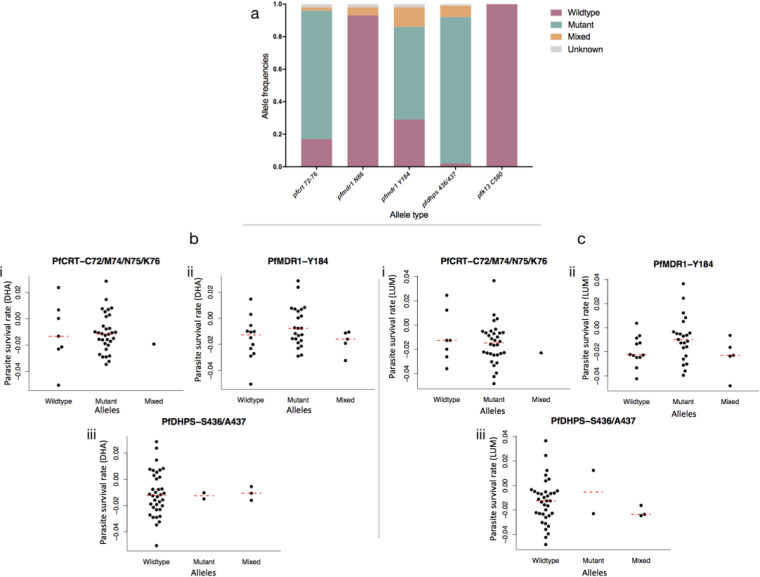
(a) Allele frequencies of 41 field isolates for the following drug resistance genes: *pfcrt* C72/M74/N75/K76, *pfmdr1* N86, *pfmdr1* Y184, *pfdhps* S436/A437, and *pfk13* C580. (b and c) Rates of parasite survival of (b) DHA and (c) LUM treatment for isolates with wild-type, mutant, and mixed alleles corresponding to *pfcrt* C72/M74/N75/K76, *pfmdr1* Y184, and *pfdhps* S436/A437. Each point in the graphs represents the parasite survival rate of an isolate. The broken red lines indicate the median survival rates of the isolates with the same alleles.

We obtained genotypes for at least 39 isolates for *pfcrt* C72/M74/N75/K76, *pfmdr1* N86, *pfmdr1* Y184, *pfdhps* S436/A437, *pfdhfr* N51/C59, and *pfk13* C580 (Table 2; see also Fig. 4). The *pfcrt* mutant haplotype was found in 79% of the isolates, with 2% mixed infections. Among those isolates, 93% were wild type for *pfmdr1* N86 and 5% mixed, while 57% were mutant for *pfmdr1* Y184 and 12% mixed. For antifolate markers, 90% of the isolates had mutation variants at *pfdhps* S436/A437 whereas all of the isolates were mutated for *pfdhfr* N51/C59. We excluded the analysis for the *pfdhfr* alleles corresponding to IT/NC as the results of scoring of the melting curves were ambiguous, showing up to 55% of mixed-allele calls. *PfK13* C580 was wild type for all isolates. Given the almost fixed frequencies of either the wild type or the mutant at these tested loci, no association with the PSRA patterns could be determined. However, for *pfmdr1* codon 184, higher LUM responses were observed for isolates with the 184F mutant allele though the mean differences between these isolates and the isolates with the Y184 wild-type variant were not significant (Fig. 4c, panel ii).

**TABLE 2:** Allele frequencies of drug resistance genes for 41 parasite isolates with drug phenotypic data (PSRA and RSA)^*a*^

Gene	Allele(s)	Codon(s)	Frequency
*pfcrt*	C72, M74, N75, K76	CMNK (wild type)	0.17
		CIET (mutant)	0.79
		CMNK/CIET (mixed)	0.02

*pfmdr1*	N86	N (wild type)	0.93
		Y (mutant)	0
		N/Y (mixed)	0.05
	Y184	Y (wild type)	0.29
		F (mutant)	0.57
		Y/F (mixed)	0.12

*pfdhps*	S436/A437	SA (wild type)	0.02
		SG (mutant)	0.88
		FG (mutant)	0.02
		SA/SG (mixed)	0.05
		FG/SA/SG (mixed)	0.02

*pfdhfr*	N51/C59	NC (wild type)	0
		IR (mutant)	0.26
		IT/NC (mixed)	
		IR/NR (mixed)	0.12
		NR/NC (mixed)	0.02

*pfk13*	C580	C (wild type)	1
		Y (mutant)	0

a *pfcrt*, P. falciparum chloroquine resistance transporter gene; *pfmdr1*, P. falciparum multidrug resistance gene 1; *pfdhps*, P. falciparum dihydropteroate synthase gene; *pfdhfr*, P. falciparum dihydrofolate reductase gene; *pfk13*, P. falciparum kelch 13 gene.

## DISCUSSION

This report describes the rates of *ex vivo* susceptibility of natural *P. falciparum* isolates from The Gambia, where transmission has declined and where we have seen increasing *ex vivo* tolerance of *P. falciparum* parasites to LUM by IC_50_ as well as modest (26%) rates of parasite survival following exposure to DHA by ring-stage survival assay (RSA) (19). These rates were obtained against DHA and LUM with a flow cytometry-based parasite survival rate assay (PSRA), with potential application to other drugs and antimalarial candidates. The potency of these drugs depends on the drug concentrations used and the length of exposure, with the assumption that cytotoxicity occurs when parasites are exposed to the active component of the drug for a prolonged time (28). Here, we used drug concentrations that are 10-fold higher than the median IC_50_ of the respective drugs obtained from the assessment of field isolates from western Gambia in 2015 (19). The use of 10-fold-higher drug concentrations, though those concentrations are much lower than serum concentrations, proved to be optimal to determine the rate of killing by slow-, medium-, and fast-acting drugs. This concentration is suboptimal, allowing a gradual effect of the drugs on the parasites (29).

The PSRA provided several advantages over the IC_50_ and RSA assays; it determines the effect of drugs over 72 h of exposure and measures both parasite growth and viability by determining reinvasion even at low parasite densities. Unlike the RSA, there is no requirement for assaying early rings, which can be difficult to obtain from natural isolates from malaria patients, thereby eliminating the need for further stressing isolates by synchronizing them with sorbitol. Similarly to the *in vivo* parasite clearance rate used for determining the efficacy of artemisinin derivatives (30), the PSRA determines clearance rates from the rate of *ex vivo* inhibition of growth over 72 h of drug exposure. This duration of exposure allows rings that emerge from tolerant isolates over the first cycle (48 h) to experience another round of drug exposure for 24 h, followed by recovery in drug-free medium. The overall outcome is the kinetics of parasite killing by the test drug over 72 h. This assay is therefore a variant of the PVFA (14, 29). Like the PVFA, the PSRA does not assess parasite metabolic activity or other parasite molecules to quantify survival or death indirectly (14). It quantifies viability from a direct count of the viable merozoites that emerge from drug-exposed schizonts and invade prestained uninfected RBCs (uRBC^DDAO-SE^). Flow cytometry provided increased sensitivity by enabling individual counting of cells and distinguishing new autologous and heterologous infected cells. With a 2-to-1 ratio of RBC^DDAO-SE^ to nonlabeled RBCs, higher numbers of prelabeled RBCs are present, skewing reinvasion to occur in these cells. As merozoites emerge after drug exposure, active reinvasion is proof of viability. This gives a good estimate of the number of parasites that survive following drug exposure. The rate of death is therefore intrinsically linked to the level of drug tolerance exhibited by each isolate. Autologous reinvasion of unlabeled RBCs is excluded from the analysis as they cannot be differentiated from dead and arrested cells. The PSRA uses a concentration of drug that is much lower than that used in the RSA but that is potent enough to kill isolates and to induce the delayed-clearance phenotype in the RSA control isolate (MRA-1241). Hence, there was a high positive correlation between PSRA and the modified flow cytometry-based RSA. With strong correlation with microscopy but improved throughput, flow cytometry-based RSA and PSRA should allow robust detection of emerging drug tolerance in natural isolates. Future and wider application of this method is warranted in Africa, where drug pressure is substantial. This is the case for The Gambia, where the artemisinin combination therapy AL is used as the first-line treatment and other ACTs are being considered for mass administration after several clinical trials.

Most of the isolates tested by PSRA in The Gambia had decreasing parasite survival with increasing days of exposure to drugs. However, four isolates exposed to DHA and three exposed to LUM continued to grow and were considered potentially tolerant, with one isolate surviving in the presence of both drugs. More isolates would have been classified as tolerant if all those that showed a rebound of growth at 72 h were included. These results suggest a state of reduced drug sensitivity, allowing parasite growth and reinvasion to occur in the presence of sublethal drug concentrations (31). The six surviving isolates could be on a path toward a persistent state of drug insensitivity that may result in resistance (32) and should be closely monitored. Extending the assay time to 96 h might also reveal clearer response profiles for the isolates with nonlinear responses over the 72-h period. Importantly, the weak correlation between initial patient parasitemia and parasite response suggests that the responses seen were not driven by the rate at which the parasites grew in the patient (*in vivo*). Most isolates had similar response patterns for the two drugs, and their survival rates correlated positively. This might be an indication of common mechanisms that enable survival of parasites following exposure to several drugs, a factor that could lead to multidrug resistance. Multidrug resistance to artemisinin derivatives and partners has been confirmed in Southeast Asia (33). We have already shown a consistent increase of LUM tolerance in The Gambia between 2012 and 2015 (19). In the same study, 26% of the isolates in the 2015 population from western Gambia showed viable parasites by microscopy-based RSA for DHA. The presence of surviving parasites in this current study, though at different proportions in the two assays, suggests a sustained low level of DHA tolerance and requires further investigation. These parasites survived and replicated under conditions of exposure to a high concentration of DHA and a prolonged period of DHA pressure with RSA and PSRA, respectively. The rate of malaria transmission in western Gambia has dropped drastically in the last decade, with prevalences of infection of lower than 5% overall and 1% for children under 5 years of age. Despite this, various ACTs remain widely available and accessible through private and public vendors. While it is officially required that ACTs should be prescribed only upon a positive malaria diagnosis, adherence to that policy is sporadic given the regularly low level of availability of rapid diagnostic test kits. We can therefore speculate that the emergence of tolerant parasites is being driven by high drug pressure against low transmission, which is hypothesized to be one of the main drivers in the emergence of antimalarial drug resistance in Southeast Asia. This calls for improved vigilance across Africa as elimination programs are implemented. ACT resistance has been shown to emerge on a backbone of known genes conferring drug resistance, including *Pfmdr1* and *Pfcrt* selected by LUM treatment. The WHO recommends surveillance for known and emerging markers of resistance in natural populations.

We genotyped the isolates assayed for alleles at *Pfmdr1*, *Pfcrt*, *Pfdhfr*, *Pfdhps*, and *Pfk13* loci that have been implicated in quinoline, antifolate, or artemisinin resistance. We found high levels of resistance loci against the antifolates, an expected result given the use of SP by SMC and IPTp. We also found high levels of *Pfmdr1* N86, the wild-type allele selected by LUM treatment, a result aligning with what we had shown before for this population (19). In contrast, the *Pfcrt* 72-76 mutant haplotype was in over 80% of isolates, indicating continuous selection by chloroquine. Approval of the use of chloroquine for treatment of malaria had been withdrawn, raising the issue of which drugs are driving selection at Pfcrt but not Pfmdr1 (34). Selection of Pfcrt may be driven by amodiaquine, which is available in combination with artesunate and accessible from private vendors in The Gambia and is the ACT of choice in neighboring Senegal (21), with whom there is significant human migration. We expect to gain more insights into this considering the current extensive temporal and spatial genome sequencing being performed for these parasite populations. Given the high levels of mutant or wild-type alleles present at drug resistance genes, an analysis of genetic association for the four different parasite PSRA profiles was not possible. However, higher survival rates against LUM were seen for isolates with the mutant variant at *Pfmdr1* 184F though the rates were not significantly different from the distribution seen in isolates with wild-type alleles. We assume that the responses observed for samples carrying multiple strains represent combined effects of the different strains present and that the six isolates that survived following exposure to either of the two drugs have specific molecular signatures influencing their phenotypes which should be further investigated. Despite the number of isolates showing growth after 72 h of exposure to DHA, no mutant alleles of *Pfk13* C580 were found in the population. Artemisinin-associated kelch 13 variants are rare in African populations, but high frequencies of other nonsynonymous single nucleotide polymorphisms (SNPs) on *Pfk13* (kelch propeller domain) had been observed for isolates from The Gambia (35). These data further buttress the idea of a need for routine and in-depth surveillance of this population.

This report highlights early signs of *ex vivo* drug tolerance of parasites from western Gambia to the most common ACT components. These signs were derived by PSRA, which provides a significant advancement in approaches implemented for the determination of parasite susceptibility. A wider application of this approach across sSA to distinguish drug tolerance and resistance will support current and future chemoprevention and chemotherapeutic strategies against malaria.

## MATERIALS AND METHODS

### Sample collection.

Ethical clearance for this study was obtained from The Gambia Government/Medical Research Council Unit in The Gambia (MRCG) Joint Ethics committee and further approved by the Gambian Ministry of Health. The study was conducted as part of a therapeutic efficacy study (TES) of AL in collaboration with the National Malaria Control Program (NMCP) at the Brikama Health Centre (Western Gambia) in 2017. Patients were included in the study following diagnosis of P. falciparum infection with a parasite density of at least 1,000/μl. Informed consent or assent was obtained from eligible patients. Two milliliters of venous blood samples were collected into EDTA tubes at day 0 of the TES and blood spots (BS) made on Whatman filter papers (Scientific Laboratory Supplies). Filter papers were air dried and stored in sealed sample bags with silica gel desiccants. Samples were transported on ice to the MRCG at the London School of Hygiene and Tropical Medicine (LSHTM) culture facility and processed within 4 h of collection.

### Parasite processing for drug assays.

Thin blood smears were made for all samples to identify parasite life cycle stages. For each sample, 50 μl was used to estimate parasite density using a C6 flow cytometer (BD Accuri; BD Biosciences) after DNA staining was performed with SYBR green I DNA intercalating dye (Applied Biosystems). To eliminate white blood cell populations from the analysis, gating was done on the red blood cell (RBC) population using only forward and side scatter parameters followed by gating of the SYBR green 1-positive population, which effectively delineates parasitized RBCs. Plasma was separated from blood cells following centrifugation for 5 min at 1,500 rpm. Equal volumes of incomplete media (RPMI 1640 [Sigma-Aldrich, United Kingdom] supplemented with 35 mM HEPES [Sigma, St. Louis, MO], 24 mM NaHCO_3_, 1 mg/liter of hypoxanthine [Sigma], and 5 μg/ml of gentamicin [Gibco-BRL]) were added to the cell pellet and layered on 6 ml of LymphoPrep (Axis Shield, United Kingdom). The layered sample was centrifuged for 20 min at 2,500 rpm, and leukocytes were aspirated. The RBCs were washed thrice by resuspending the pellet in incomplete media and centrifuged for 5 min at 1,500 rpm. The washed pellet was resuspended in growth medium (incomplete medium with 0.5% AlbuMAX [Gibco-BRL]). The parasitemia was normalized to 0.5% (1,000 parasites/μl) for all samples with parasitemia levels higher than 0.5% and 2% hematocrit using uninfected type O-positive (O^+^) heterologous RBCs prior to PSRA and RSA. Four laboratory-adapted strains (3D7, Dd2, and MRA-1239, which are sensitive to both LUM and DHA, and MRA-1241, which is sensitive to LUM but resistant to DHA) were used as internal controls. The isolates were routinely cultured with fresh O^+^ RBCs and maintained at 2% hematocrit with growth media under standard incubation conditions of 37°C, 90% N_2_, 5% O_2_, and 5% CO_2_. All laboratory-adapted strains were synchronized twice with 5% d-sorbitol to obtain ≥80% ring stages prior to assay setup. A total of 170 samples were obtained from Brikama Health Centre in 2017.

### Parasite survival rate assay (PSRA).

The parasite survival rate assay is based on reinvasion of surface-labeled uninfected O^+^ RBCs (uRBC) by merozoites emerging from ruptured schizonts that developed after drug exposure of infected samples. This represents a modification of a previously described protocol (14). Here, target uRBCs were prelabeled with the amine-reactive fluorescent dye 7-hydroxy-9H-(1,3-dichloro-9,9-dimethylacridin-2-one) succinimidyl ester (DDAO-SE; Invitrogen) (10 μM), a Far Red cell dye, as previously described (36). A 2% hematocrit suspension of the uRBCs in incomplete media with 10 μM DDAO-SE was made and incubated at 37°C for 2 h with shaking. The suspension was washed once, resuspended with incomplete media, and reincubated for a further 30 min. Suspensions of DDAO-SE-labeled uRBC (uRBC^DDAO-SE^) were washed and reconstituted with growth media to achieve a final hematocrit of 2%.

**FIG 5:**
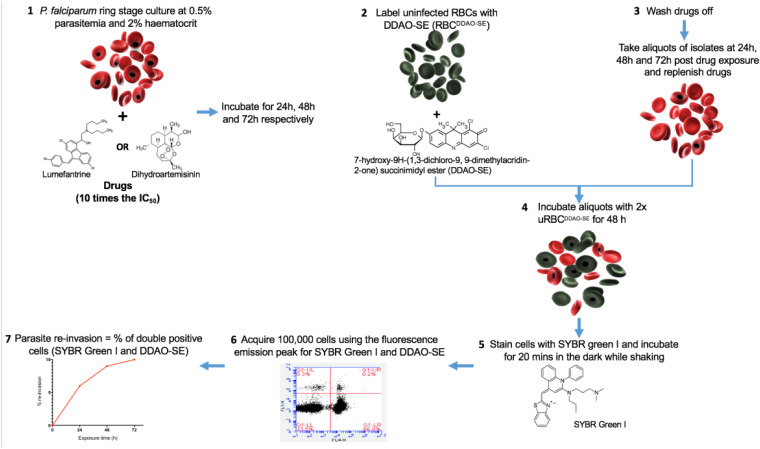
Schematic representation of *ex vivo* parasite survival rate assay. (Step 1) iRBCs at 0.5% parasitemia and 2% hematocrit are incubated with 10-fold median IC_50_s of dihydroartemisinin and lumefantrine for 24, 48, and 72 h. (Step 2) uRBCs are labeled with the intracellular dye DDAO-SE (uRBC^DDAO-SE^). (Step 3) Drugs are washed off from the reaction mixtures from step 1 every 24 h, aliquots taken, and drugs replenished. (Step 4) Postexposure drug-free aliquots are incubated with 2× uRBC^DDAO-SE^ for a further 48 h. (Steps 5 and 6) The aliquots are then counterstained with SYBR green I for flow cytometric analysis (step 5), and 100,000 cells are acquired (step 6). (Step 7) Doubly positive stained cells are analyzed.

PSRAs were set up using laboratory isolates of ≥80% rings (*in vitro*) and field isolates within 4 h of sample collection (*ex vivo*). The assays were done in triplicate to determine levels of sensitivity to concentrations that were 10-fold higher than the median IC_50_ of the respective drugs determined in a previous study (19). Briefly, 100 μl of parasite suspension at 0.5% parasitemia and 4% hematocrit was added to 48-well microtiter plates precoated with 100 μl of the corresponding drugs at twice the target concentration [(10-fold median IC_50_) × 2]. This resulted in a final drug concentration of 10-fold median IC_50_ of DHA (8.1 nM) and LUM (398 nM) at 2% hematocrit. A no-drug control, substituted with 0.1% dimethyl sulfoxide (DMSO [Sigma-Aldrich, United Kingdom]), was assayed for each sample. The samples in the microtiter plates were incubated for 24, 48, or 72 h under standard incubation conditions. Drugs were refreshed every 24 h after washing the cells by incubating twice for 5 min with incomplete media. Fifty microliters of the drug-free suspension was transferred to a fresh 96-well microtiter plate containing 100 μl of uRBC^DDAO-SE^ (1-in-3 dilution), which was further incubated for 48 h (Fig. 5).

Each sample was then washed and counterstained with a 1:10,000 dilution of SYBR green I–phosphate-buffered saline (PBS). For this, 100 μl of diluted stain solution was added to each assay well of the microtiter plate and incubated in the dark at room temperature with shaking for 20 min. Stained cells were washed twice and resuspended with 200 μl of PBS. A further 1-in-4 dilution with PBS was done prior to flow cytometry counting using a BD Accuri C6 flow cytometer. For acquisition, the fluorescence emission peaks for SYBR green I and DDAO-SE were set at 520 nm and 657 nm for the green and red channels, respectively. For each assay well, 100,000 events were acquired, and data were analyzed using BD Accuri C6 software.

### Ring-stage survival assay (RSA).

A modification of the RSA protocol (12, 37) was carried out to assess the reinvasion potential of parasites exposed to 700 nM DHA, replacing microscopy with two-color flow cytometry similarly to the PSRA protocol described above. Leukocyte-depleted infected RBCs (iRBCs) were set up in duplicate at 0.5% parasitemia and 2% hematocrit. Each isolate was exposed to 700 nM DHA and 0.1% DMSO as a control for exactly 6 h under standard incubation conditions. DHA and DMSO were then washed off using incomplete media and parasites resuspended with drug-free growth media. Fifty microliters of this suspension was added to 100 μl uRBC^DDAO-SE^ in a separate 96-well microtiter plate, and both plates were incubated for a further 66 h. Thin blood smears were then made and stained with Giemsa following the standard RSA protocol. Ring-stage survival rates were determined microscopically using the initial parasitemia before drug exposure (initial parasitemia: INI), the DMSO control (nonexposed [NE]), and DHA exposure (DHA). Ring-stage survival was calculated for isolates with a growth rate of ≥1% using the following published formula:

Percent survival (%) = (DHA/NE)× 100

The cells incubated with uRBC^DDAO-SE^ were counterstained with SYBR green I as described above and acquired using a BD Accuri C6 flow cytometer to determine parasite reinvasion rates.

### Genotyping of selected drug resistance loci.

Genotyping was done by locus-specific high-resolution melting (HRM) assays with parasite DNA extracted from filter-paper-dried blood spots (DBS). To recover parasite DNA, DBS were punched onto plates containing 96 deep wells, using punchers and forceps that were rinsed in 1% bleach and alpha-Q water after each sampling step to limit cross-contamination. For each plate, 4 negative and 4 positive controls were included. Genomic DNA (gDNA) was manually extracted using a QIAamp 96 DNA blood kit (Qiagen, Hilden, Germany) and the manufacturer’s instructions. The DNA concentrations of the eluates were quantified using a NanoDrop 1000 spectrophotometer (Thermo Scientific) and stored at –20°C until use. One micromolar volume of gDNA of approximately 10 pg (1 ng/μl) was used for genotyping assays. HRM genotyping reactions were performed for alleles *pfcrt* C72/M74/N75/K76, *pfmdr1* N86, *pfmdr1* Y184, *pfdhps* S436/A437, *pfdhfr* N51/C59, and *pfk13* C580 on a LightCycler 96 real-time PCR system (Roche). The primers and probes used for PCRs with a 2.5× LightScanner master mix (Biofire) were as previously described (38). Each reaction mixture had final forward and reverse primer concentrations of 0.05 μM and 0.2 μM, respectively (asymmetric PCR) and a 0.2 μM concentration for allele-specific probes. The PCR conditions and analysis method used were as previously described (38).

### Statistical analysis of drug survival rates.

The analysis was aimed mainly at evaluation of the effects of drug exposure by determination of the magnitude of the decline in growth caused by reinvasion according to the time of exposure compared to drug-free controls, i.e., LUM-treated isolates versus no-drug control and DHA-treated isolates versus no-drug control. We also aimed to explore patterns of variation in response to drug exposure between isolates. To first assess the effect of time of exposure on survival (growth), the reinvasion rate values were log-transformed for normality and the resulting values were used to generate descriptive statistics representative of the responses at each time point. Linear mixed-effect models were then fitted to examine the heterogeneity in drug susceptibility, allowing for interaction between time and drug treatment group (LUM or DHA) with random effects on subjects. Since there were three discrete time points of measurement, with the data serving as an indication of a potential nonlinear relationship between treatment response and time, we used time as a categorical variable to determine differences in the effects of treatment performed for 48 and 72 h against 24 h as the reference. As such, we were able to examine the effect of longer exposure. To explore the differences in susceptibility to each drug per isolate, we first determined the drug effect from analysis of the difference between drug-exposed isolates and no-drug control isolates at each time point. We then fitted a linear trend across time points for each isolate, deriving patterns of individual growth decay slopes. These estimated slopes represent individual parasite survival (or death) rates. On the basis of the derived decay patterns (individual trajectories), an isolate was assigned to one of the following four classes: linear decrease (−−−), linear increase (+++), nonlinear increase/decrease (+−+) and nonlinear decrease/increase (−+−). In addition, we assessed the relationship between individual trajectories and their corresponding genotypes. All analyses were performed using the R package (RStudio version 1.2.5001) and Stata 14 (StataCorp, College Station, TX, USA). A *P* value of <0.05 was considered significant. Other plots were explored using Prism (GraphPad Prism version 7.0a).

## Supplementary Material

Supplemental file 1
