# Supplementary material for: Tolerance of Gambian Plasmodium falciparum to Dihydroartemisinin and Lumefantrine Detected by Ex Vivo Parasite Survival Rate Assay
Source: Antimicrob Agents Chemother. 2020 Dec 16;65(1):e00720-20. doi: 10.1128/AAC.00720-20 (PMC7927851; doi:10.1128/AAC.00720-20)
Supplement: Supplemental file 1 [file AAC.00720-20-s0001.pdf]

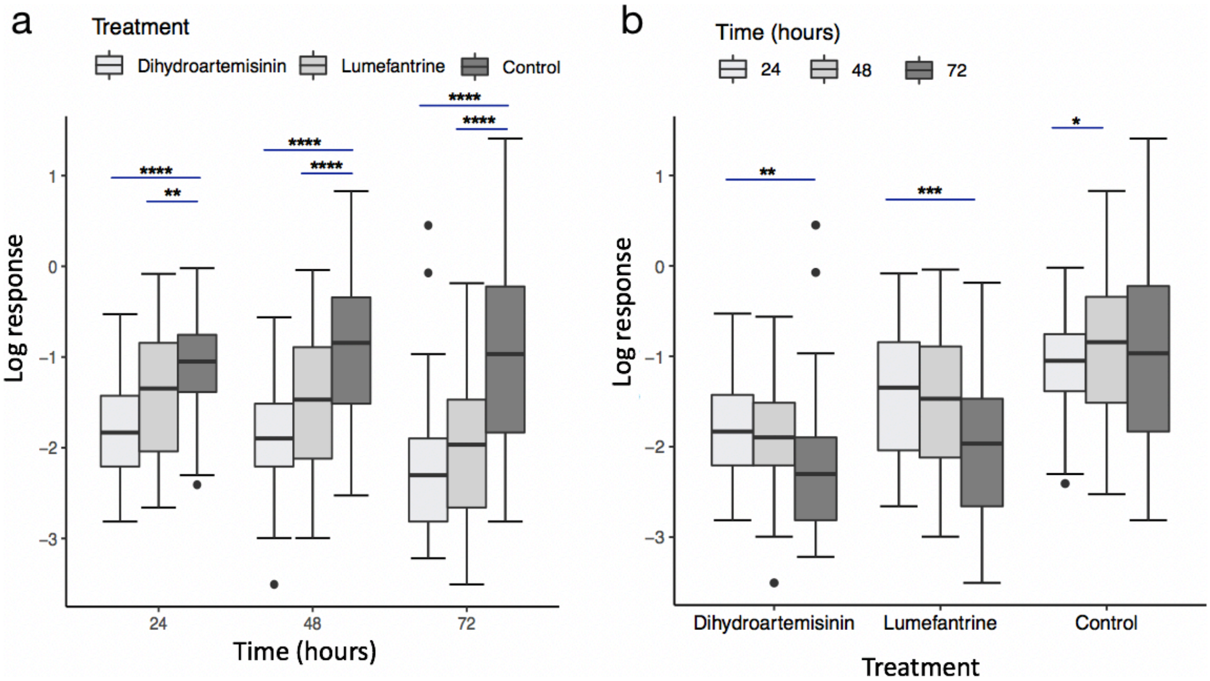

Supplementary figure 1. Parasite log responses following exposure to Dihydroartemisinin, Lumefantrine and DMSO-treated control at 24-, 48- and 72- hours of drug exposure with PSRA. Mixed model for log response and interaction between group and time with random intercept was fitted. Mean responses comparing the log response of (a) the three treatment groups at each timepoint and (b) the three timepoints for each treatment groups. $p < 0.05$ was considered significant.

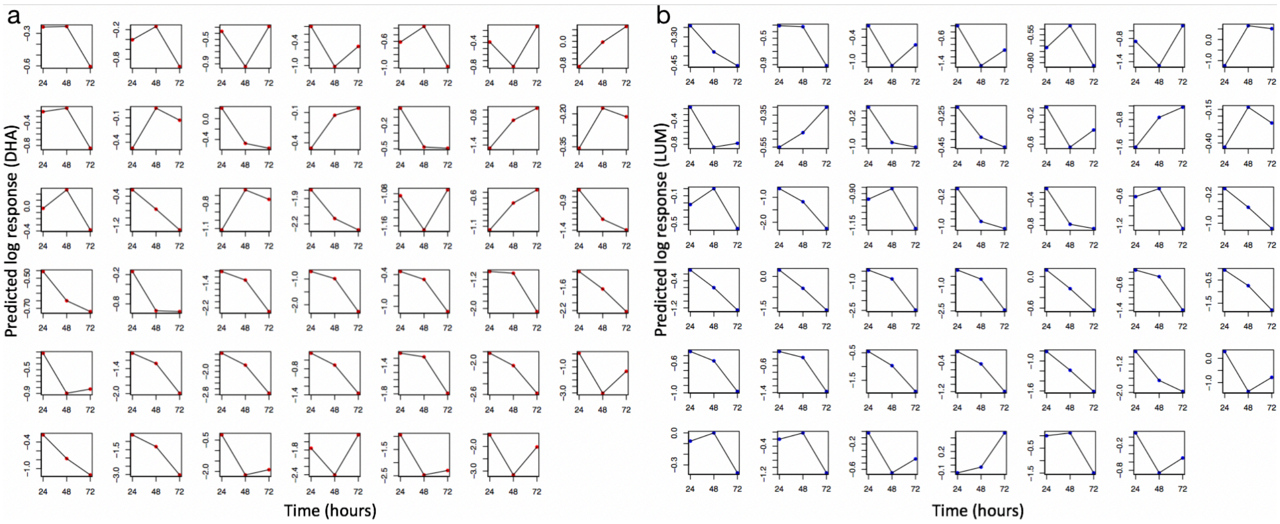

Supplementary figure 2. Individual profiles of 41 isolates following exposure to (a) DHA and (b) LUM at 24-, 48- and 72- hours with PSRA. Each point on the individual plots represent the difference between the predicted response of the DMSO-treated control and drug treatment. The connecting lines give an indication of the response pattern of each isolate.

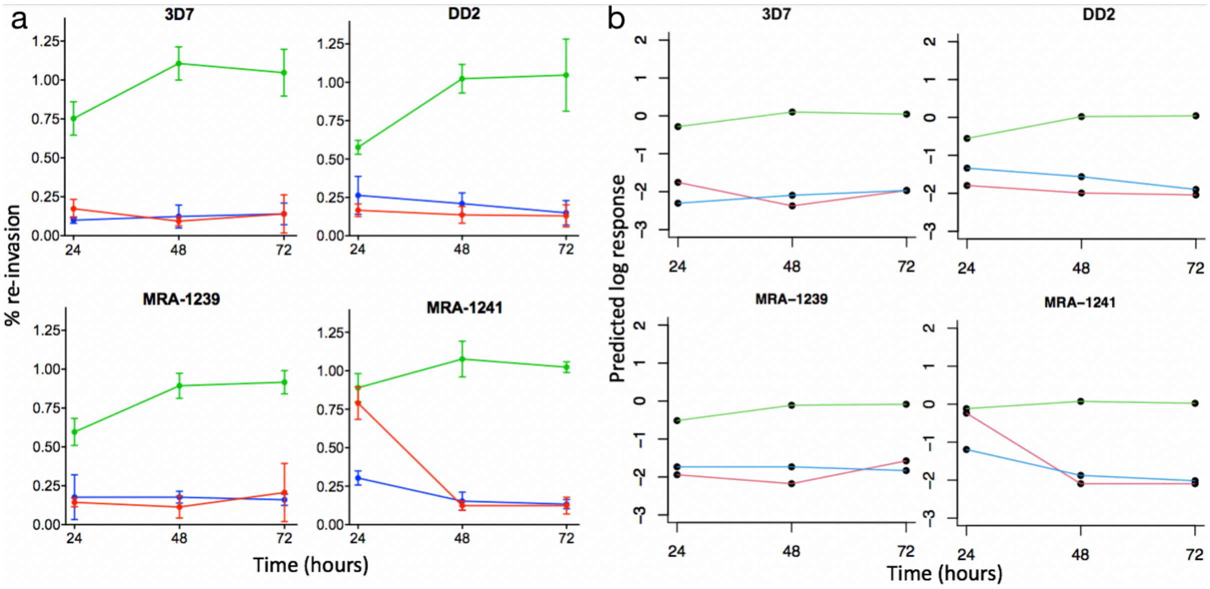

Supplementary figure 3. (a) Re-invasion parasitemias and (b) log response rates from mixed model analysis of 4 lab adapted isolates following exposure to DHA (red lines), LUM (blue lines) and DMSO-control (green lines) at 24-, 48- and 72- hours with PSRA. In (a), the confidence intervals of % re-invasions for 3 replicates of 3 independent experiments are shown in bars.

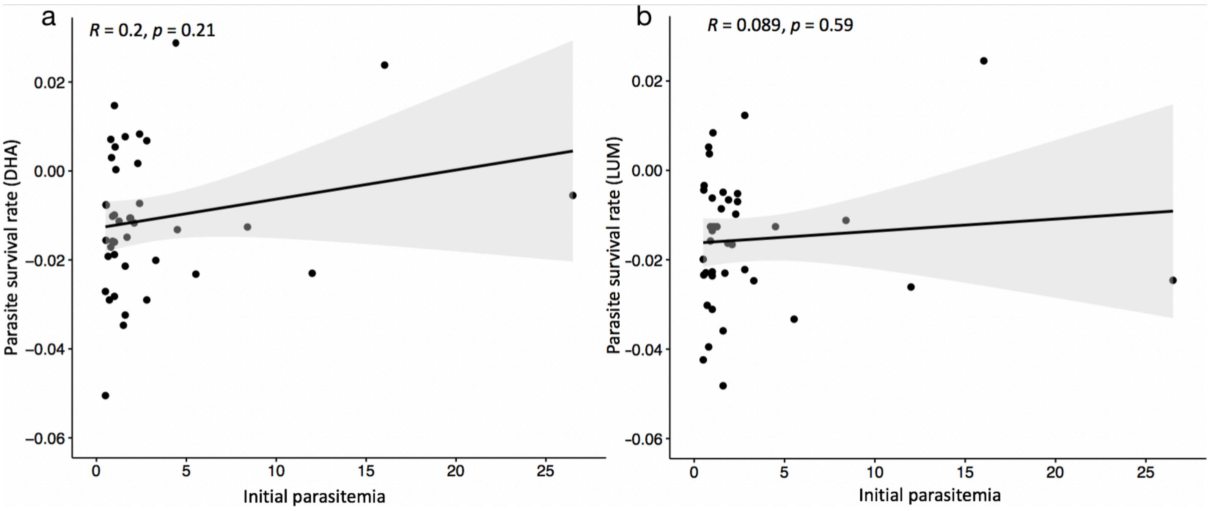

Supplementary figure 4. Correlation analysis between initial patient parasitemia at day 0 prior to assay set-up and parasite survival rates of isolates treated with (a) DHA with a Pearson correlation coefficient of R = 0.2 and p = 0.21 and (b) LUM with R = 0.089 and p = 0.59.
